# Relationship between effective lens position and axial position of a thick intraocular lens

**DOI:** 10.1371/journal.pone.0198824

**Published:** 2018-06-14

**Authors:** Simon Schröder, Achim Langenbucher

**Affiliations:** Institute of Experimental Ophthalmology, Saarland University, Homburg, Saarland, Germany; Bascom Palmer Eye Institute, UNITED STATES

## Abstract

**Purpose:**

To discuss the impact of intraocular lens-(IOL)-power, IOL-thickness, IOL-shape, corneal power and effective lens position (ELP) on the distance between the anterior IOL vertex (ALP) of a thick IOL and the ELP of its thin lens equivalent.

**Methods:**

We calculated the ALP of a thick IOL in a model eye, which results in the same focal plane as a thin IOL placed at the ELP using paraxial approximation. The model eye included IOL-power (P), ELP, IOL-thickness (Th), IOL-shape-factor (X), and corneal power (DC). The initial values were P = 10 D (diopter: 1 D = 1 m^-1^), 20 D, 30 D, Th = 0.9 mm, ELP = 5 mm, X = 0, DC = 43 D. The difference between ALP and the ELP was illustrated as a function of each of the model parameters.

**Results:**

The ALP of a thick lens has to be placed in front of the ELP for P>0 IOLs to achieve the same optical effect as the thin lens equivalent. The difference ALP-ELP for the initial values is -0.57 mm. Minus power IOLs (ALP-ELP = -0.07 mm, for IOL-power = -5 D) and convex-concave IOLs (ALP-ELP = -0.16 mm, for X = 1) have to be placed further posterior. The corneal power and ELP have less influence, but corneal power cannot be neglected.

**Conclusion:**

The distance between ELP and ALP primarily depends on IOL-power, IOL-thickness, and shape-factor.

## Introduction

The estimation of the postoperative intraocular lens (IOL) position based on preoperative measurement is the largest source of uncertainty for the choice of the appropriate IOL-power [1]. Most IOL power calculation formulae assume that the IOL can be represented as a thin lens that is placed at the effective lens position (ELP) behind the cornea [2–5]. The prediction of the ELP is generally not identical with the position of a real IOL inside the eye [6], neither with the front vertex nor the back vertex or the secondary principal plane. Insights into the relationship between ELP and the actual position of the thick IOL can help to improve and refine IOL-power calculation formulae [7].

Postoperatively, the ELP cannot be directly measured. Instead, it is recalculated from the refractive outcome after surgery and the constants of the IOL formulae are refined based on a high number of cases. The recalculation suffers from the uncertainty of the measurements [8]. Modern biometers are able to directly measure the position of the anterior surface of the IOL (anterior lens position, ALP). Establishing relationships between the ELP and anterior IOL surface position thus enables another way of estimating the ELP based on direct measurement of the IOL position in the pseudophakic eye [7], which does not suffer from the statistical uncertainty of the corneal power and postsurgical refraction measurement.

The prediction of the real IOL position in the pseudophakic eye can be useful for the ray-tracing assisted choice of the IOL with respect to the refractive outcome and higher order aberrations [9], if IOL- manufacturers are willing to provide shape information for their IOLs. For most IOL models there is no optimized prediction of the IOL position available, although calculation approaches for the thick lens position exist [10]. As a starting point, the IOL position of the thick IOL might be estimated from the ELP.

This manuscript discusses the dependence of the distance between ALP and ELP on IOL-power, IOL-thickness, IOL-shape, corneal power, and ELP.

## Materials and methods

Paraxial matrix optic was used with a simplified eye-model to study the impact of IOL-power, IOL-thickness, IOL-shape, corneal power and ELP on the distance between the ALP and the ELP (S1 Supporting Information). The model consists of cornea, anterior chamber IOL and vitreous. Corneal power was modeled as a thin lens. The refractive index of the eye was 1.336 [11]. For the IOL, we exemplarily assumed a hydrophilic IOL with a refractive index of 1.46. First, the axial length was calculated with a thin IOL placed at the ELP. The axial length was defined as the distance of the focus behind the cornea. Then, the thin lens was replaced by a thick IOL of identical refractive power (P) and the ALP that resulted in the same axial length was calculated.

The initial values of the eye model were: corneal power *DC* = 43 D (diopter 1 D = 1 m^-1^), ELP = 5 mm, IOL-thickness Th = 0.9 mm, shape-factor X = 0. The Coddington shape-factor X is given by X = (*Rp+Ra*)/(*Rp-Ra*), where *Ra* is the anterior radius and *Rp* the posterior radius of the IOL. Realistic IOL models shall have 0<*X*<1 [12]. A shape-factor of -1<*X*<1 indicates a biconvex lens, X>1 a convex-concave lenses (with the convex side facing anteriorly), and X<-1 a concave-convex lens. The calculations was performed for IOL-power of 10 D, 20 D, and 30 D. The dependence of the difference between ALP and ELP on any of the model’s parameters was illustrated with the remaining parameters kept at their initial values.

## Results

**Fig 1:**
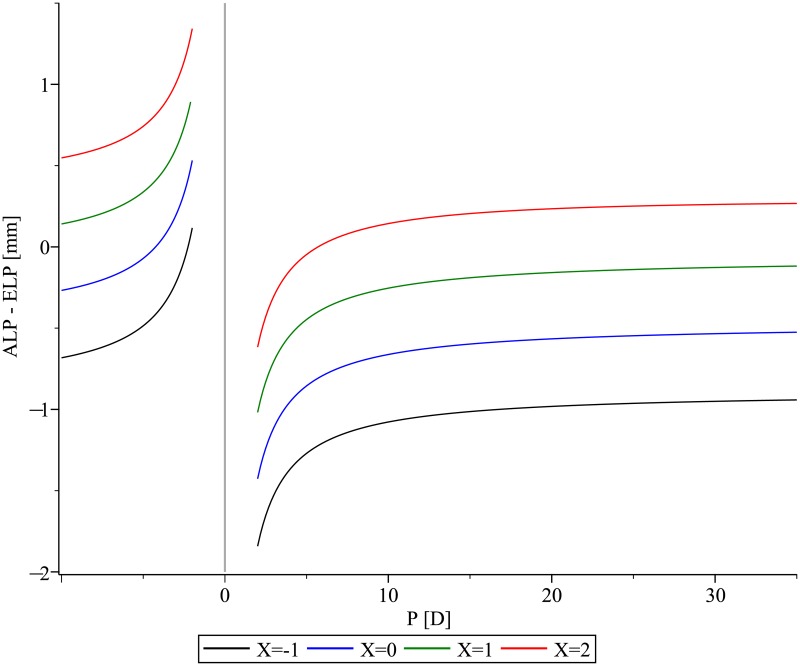
The difference between the anterior intraocular lens (IOL) vertex and the effective lens position of the thin lens equivalent as a function of IOL-power for IOLs with three different shape-factors X = -1, 0, 1, 2.

The difference ALP and ELP strongly depends on the power of the IOL (Fig 1, Table 1). Negative powered IOLs have to be placed more posteriorly than positive powered IOLs, if the same ELP is assumed.

**Table 1:** Difference between the anterior intraocular lens vertex and the effective lens position of the thin lens equivalent for different model parameters. The initial settings are highlighted in yellow, the parameters that were varied are highlighted in green.

**P [D]**	20	-5	20	20	20	20
**Th [mm]**	0.9	0.9	0	0.9	0.9	0.9
**X**	0	0	0	1	0	0
**DC [D]**	43	43	43	43	50	43
**ELP [mm]**	5	5	5	5	5	3
**ALP—ELP [mm]**	-0.57	-0.07	0	-0.16	-0.58	-0.56

P: IOL-power, Th: IOL-thickness, X: Coddington shape-factor, DC: corneal power, ELP: effective lens position

**Fig 2:**
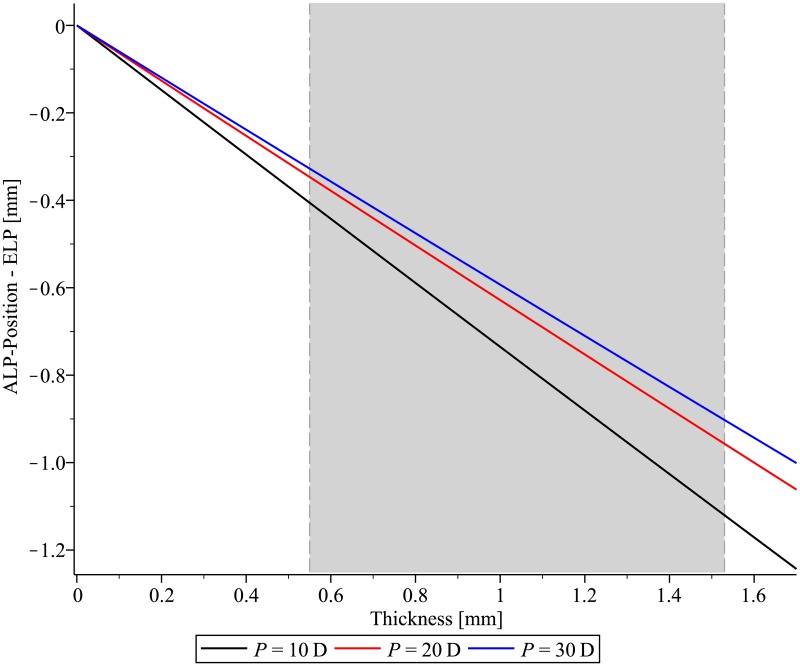
The difference between the anterior intraocular lens (IOL) vertex and the effective lens position of the thin lens equivalent as a function of IOL-thickness for IOLs with optical power P = 10 D, 20 D, 30 D. The clinically relevant range of IOL-thickness is marked in grey.

**Fig 3:**
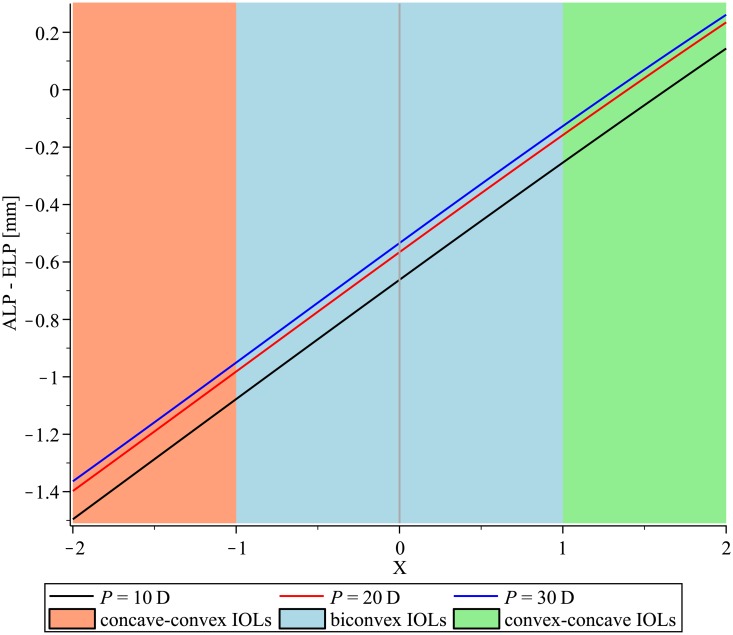
The difference between the anterior intraocular lens (IOL) vertex and the effective lens position of the thin lens equivalent as a function of the shape-factor for IOLs with optical power P = 10 D, 20 D, 30 D.

If the IOL-thickness is set to zero, the ELP has to be identical to the distance between cornea and anterior IOL (Fig 2). The distance between ALP and ELP becomes larger with increasing IOL-thickness, and the ALP has to be placed in front of the ELP to achieve the same optical effect as the thin lens. The ALP has to be placed more posteriorly with increasing shape-factor X (Fig 3).

**Fig 4:**
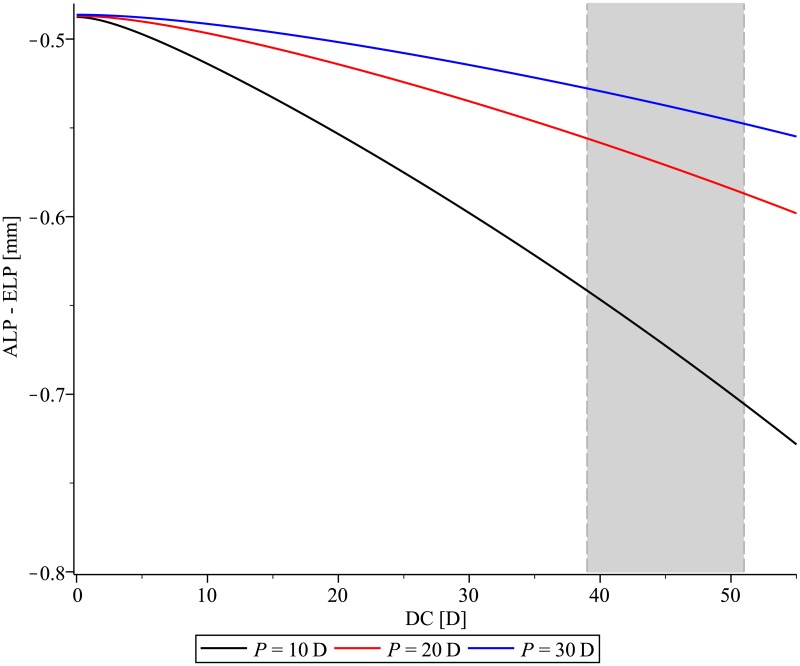
The difference between the anterior intraocular lens (IOL) vertex and the effective lens position of the thin lens equivalent as a function of corneal power (DC) for IOLs with optical power P = 10 D, 20 D, 30 D. The clinically relevant range of corneal power is marked in grey.

The optical power of the cornea results in increased distance between ALP and ELP (Fig 4). The IOL had to move anteriorly with increasing corneal power in order to focus to the same image plane as a thin lens placed at ELP. This effect was larger for the 10 D IOL than for the 20 D or 30 D IOL.

**Fig 5:**
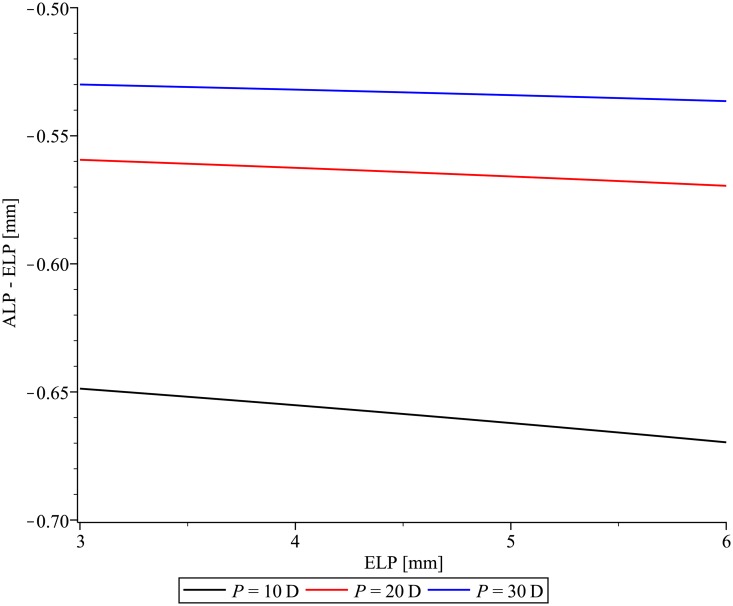
The difference between the anterior intraocular lens (IOL) vertex and the effective lens position (ELP) of the thin lens equivalent as a function of ELP for IOLs with optical power P = 10 D, 20 D, 30 D.

The ELP has a minor effect on the difference between ALP and ELP (Fig 5). For the initial parameter values, this difference changes by less than 0.02 mm for a P = 20 D IOL placed at ELP = 2 mm or ELP = 6 mm.

## Discussion

Identical ELP values do not correspond to identical positions of a thick IOL inside the pseudophakic eye. The difference between ELP and ALP primarily depends on IOL-power (Fig 1), IOL-thickness (Fig 3), and shape-factor (Fig 4).

This effect is most pronounced when comparing negative to positive powered IOLs. An IOL with 0 D optical power has no optical effect, if the IOL is modeled as a thin lens. Consequently, for a given ELP, its ALP was undefined.

With increasing IOL-thickness, the distance between the ALP and the principal plane increases (for P>0 IOLs) resulting in an IOL that must be placed more anteriorly to the ELP. For vanishing IOL-thickness, the calculation of a thick lens turns into its thin-lens equivalent and the IOL position has to be identical to the ELP regardless of the IOL-power or shape. The thickness increases linearly wit IOL-power for P = 10 D to 30 D [13]. In the range P = 10 D to 30 D, the impact of thickness and IOL-power on the difference between the ELP and the position of the thick lens should partially compensate for each other.

If there was no cornea, the thick IOL had to be placed with its principal plane at the ELP to achieve the same focal length as the thin lens equivalent. Due to the positive refractive power of the cornea, the IOL has to be placed anteriorly [7]. It is insufficient to place a thick IOL with its principal plane at the ELP for ray-tracing simulations of the pseudophakic eye with a thick IOL. The difference between the ALP and ELP (IOL with P = 20 D) varies by less than 0.03 mm as a function of DC for the normal range of DC (between 40 D and 51 D [14]). The corneal power has larger impact for thick low powered IOLs.

The difference between ALP and ELP is strongly affected by the refractive index. With a refractive index of 1.52 the ALP is decreased by approximately 0.07 mm compared to the ALP with the initial settings.

Paraxial ray transfer matrix methods are well established for IOL calculations [15–17]. Ray transfer matrix methods can neither take non-paraxial rays into account nor consider the effect of asphericity of the cornea or IOL. Ray transfer matrices can be used as first order approximations to estimate the ALP for ray-tracing simulations based on the ELP that was calculated from optimized IOL-constants.

The reverse way is to calculate the ELP based on the ALP. Good agreement between the standard approach of estimating the ELP based on postoperative refraction and corneal power and the calculation from the direct ALP measurements has been observed [7]. Whereas the first biometers where not able to measure the IOL-position, modern day optical coherence (OCT) technology can measure the ALP with good repeatability [18]. The calculation of the ELP based on the measured ALP could remove some uncertainty from IOL-constant optimization, which come from the statistical uncertainty of the corneal power and postoperative refraction measurement used to estimate ELP [8]. However, the ELP values based on the pseudophakic ALP might be different from the standard approach, because of the aberration of the eye and systematic measurement errors.

Improvement of IOL-power prediction formulae might be possible by taking IOL-shape, IOL-thickness, the IOL-materials’ refractive index, and IOL-power into account, because they strongly affect the difference between ELP and ALP. At least, IOL constants should be optimized separately for negative and positive powered IOLs. IOL-manufacturers could assist to make conventional ELP estimation more reliable by manufacturing IOLs in a way that the difference between the haptic plane and the ELP varies only minimally with IOL-power.

The shape of the IOL is usually biconvex [19], but low powered IOLs might be designed with a convex anterior surface and a concave posterior surface (X>1). The difference between ELP and ALP from the equiconvex IOL (X = 0) to the convex-concave meniscus IOL (X = 2) was around 0.8 mm for a P = 10 D IOL. If the IOL-manufacturers provide the respective IOL-power where the shape switches from biconvex to a meniscus lens design, this information could be included in modern IOL-formulae and IOL-constant-optimization to improve ELP and ALP predictions.

In conclusion, the anterior IOL surface of a thick lens has to be placed in front of the ELP for positive powered IOLs to achieve the same optical effect as the thin lens equivalent. The distance between ELP and anterior IOL surface strongly depends on IOL-power, IOL-thickness, and IOL-shape. The corneal power and ELP have less influence, but corneal power cannot be neglected.

## Supporting information

S1 Supporting Information Print-out of the calculation sheet (algebra software: Maple 2017) used to study the difference ALP-ELP based on the ray-matrix method. (PDF)
