# Supplementary material for: Relationship between effective lens position and axial position of a thick intraocular lens
Source: PLoS One. 2018 Jun 14;13(6):e0198824. doi: 10.1371/journal.pone.0198824 (PMC6001946; doi:10.1371/journal.pone.0198824)
Supplement: S1 Supporting Information — (PDF) [file pone.0198824.s001.pdf]

*restart* :
*with*(*plots*) : *with*(*plottools*) :

# Calculate Thick-lens-position from (classical) ELP as a function of: corneal power, IOL-thickness, or shape factor

**Define the Transfer-matrices for the thin-lens placed at the effective lens position (ELP) case:**

Lane distance to Cornea = Infinity:

Transmission / refraction matrices for the optical elements.
Refraction, cornea:

$$mRefractCornea := \begin{bmatrix} 1 & 0 \\ -\frac{DC}{nEye} & 1 \end{bmatrix}:$$

Transmission through ELP:

$$mTransELP := \begin{bmatrix} 1 & ELP \\ 0 & 1 \end{bmatrix}:$$

Refraction, thin IOL:

$$mRefractIOLthin := \begin{bmatrix} 1 & 0 \\ -\frac{PIOL}{nEye} & 1 \end{bmatrix}:$$

Transmission from thin IOL to retina:

$$mTransVitreousELP := \begin{bmatrix} 1 & AL - ELP \\ 0 & 1 \end{bmatrix}:$$

**Define the Transfer-matrices for the thick-lens case:**

(Refraction through cornea is identical to *mRefractCornea*)

Transmission through ALP:

$$mTransELPa := \begin{bmatrix} 1 & ELPa \\ 0 & 1 \end{bmatrix}:$$

Calculate the IOL-Radii as a function of Coddington Shape-factor X from the IOL-power PIOL,
PIOLThick is the IOL-power according to the lens-maker's equation (for thick lenses)
*Ra* = anterior radius, *Rb* = poserior radius

$$Rb := \frac{Ra \cdot (1 + X)}{(X - 1)} :$$

$$PIOLThick := -(nEye - nLens) \cdot \left( \frac{1}{Ra} - \frac{1}{Rb} - \frac{(nEye - nLens)}{nLens} \cdot \frac{Thick}{Ra \cdot Rb} \right) :$$

$$R := solve(PIOLThick - PIOL = 0, Ra) :$$

$Ra := R[2]$ : Refraction, anterior IOL:

$$mRefractIOLA := \begin{bmatrix} 1 & 0 \\ \frac{nEye - nLens}{Ra \cdot nLens} & \frac{nEye}{nLens} \end{bmatrix}:$$

Transfer through IOL:

$$mTransIOL := \begin{bmatrix} 1 & Thick \\ 0 & 1 \end{bmatrix}:$$

Refraction, posterior IOL:

$$mRefIOLP := \begin{bmatrix} 1 & 0 \\ \frac{nLens - nEye}{Rb \cdot nEye} & \frac{nLens}{nEye} \end{bmatrix}:$$

Transfer through posteriorIOL-->retina:

$$mTransVitreousELPb := \begin{bmatrix} 1 & AL - ELPa - Thick \\ 0 & 1 \end{bmatrix}:$$

---------------------------------------------------------------------------------------------------------

**Calculate the ALP as a function of effective lens position ELP, corneal power DC, IOL-thickness, Shape factor X**

Calculate the transfer-matrix and image-position (AL) with the thin lens formula:
$mTransferThinIOL := mTransVitreousELP \cdot mRefractIOLthin \cdot mTransELP \cdot mRefractCornea$ :
$AL := (solve(mTransferThinIOL(1, 1) = 0, AL))$ :

Calculate the transfer-matrix and ALP with the thick lens formalism:
$mTransferThickIOL := mTransVitreousELPb \cdot mRefIOLP \cdot mTransIOL \cdot mRefractIOLA \cdot mTransELPa$
$\cdot\, mRefractCornea$ :
$ALP := solve(mTransferThickIOL(1, 1, ) = 0, ELPa)$ :

<u>Set the initial parameters:</u>

set refractive indices:
$nEye := 1.336$ :
$nLens := 1.46$ :

$Thick := \frac{0.9}{1000}$ :
$DC := 43$ :
$PIOL := 20$ :
$ELP := \frac{5}{1000}$ :

---------------------------------------------------------------------------------------------------------

**Calculate ELP-ALP as a function of shape factor for IOL power P = 10 D, 20 D, and 30 D**
(example for the Fig. 1-5 that show the dependence of ALP-ELP on the different model parameters)

```
ELP2 := unapply(ALP[1], X) :
p1 := plot(1000·(ELP2(X) − ELP), X=-2 ..2, color = "Red", size = [600, 500]) :

unassign('PIOL') : PIOL := 10 :
ELP25 := unapply(ALP[1], X) :
unassign('PIOL') : PIOL := 10 : ELP20 := unapply(ALP[1], X) : p2 := plot(1000·(ELP20(X)
    − ELP), X=-2 ..2, color = "Black") : unassign('PIOL') : PIOL := 30 : ELP21 :=
    unapply(ALP[1], X) : p3 := plot(1000·(ELP21(X) − ELP), X=-2 ..2, color = "Blue") : p4 :=
    plot([0, y, y=-1.51 ..0.3], color = "DarkGrey") : p5 := plot([-1, y, y=-1.51 ..0.3], color
    = "DarkGrey", linestyle = "dash") : p6 := plot([1, y, y=-1.51 ..0.3], color = "DarkGrey") :
p7 := shadebetween(-1.51, 0.3, x=-2 ..-1, color = "LightSalmon", transparency = 0.3) :
p8 := shadebetween(-1.51, 0.3, x=-1 ..1, color = "LightBlue", transparency = 0.3) :
p9 := shadebetween(-1.51, 0.3, x = 1 ..2, color = "LightGreen", transparency = 0.3) :

display([p7, p8, p9, p2, p1, p3, p4], overrideoptions, axes = frame, labels = ["X", "ALP - ELP [mm]"],
    labeldirections = [horizontal, vertical], legend = ["concave-convex IOLs", "biconvex IOLs",
    "convex-concave IOLs", P = 10 D, P = 20 D, P = 30 D, ""])
```

# IOL-Position vs. Shape-factor

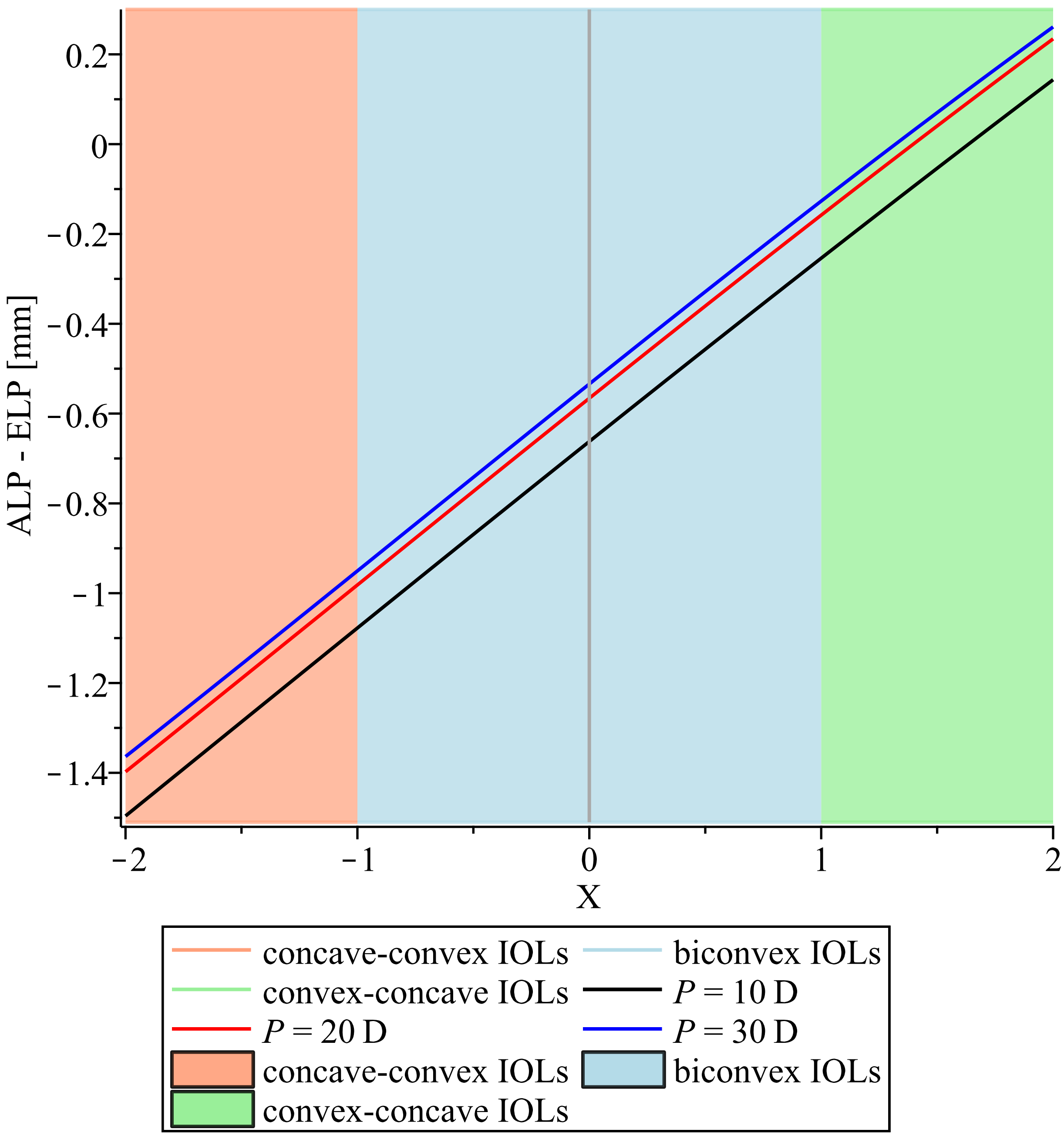


## IOL-Position vs. n(IOL) (X=0)

```
unassign('nLens') :
 unassign('PIOL') :
 PIOL := 20 : X := 0 :
 ELPnLens20 := unapply(ALP[1], nLens) :
p1 := plot(1000·(ELPnLens20(nLens) − ELP), nLens = 1.4 ..1.6, color = "Red", size = [600, 500]) :
 unassign('PIOL') : PIOL := 10 :
ELPnLens10 := unapply(ALP[1], nLens) :
 p2 := plot(1000·(ELPnLens10(nLens) − ELP), nLens = 1.4 ..1.6, color = "Black") :
 unassign('PIOL') : PIOL := 30 :
 ELPnLens30 := unapply(ALP[1], nLens) :
```

$p3 := plot(1000 \cdot (ELPnLens30(nLens) - ELP), nLens = 1.4 ..1.6, color = "Blue") :$
$display([p2, p1, p3], overrideoptions, axes = frame, labels = ["n(IOL)", "ALP - ELP [mm]"],$
$labeldirections = [horizontal, vertical], legend = [P = 10 \text{ D}, P = 20 \text{ D}, P = 30 \text{ D}]);$

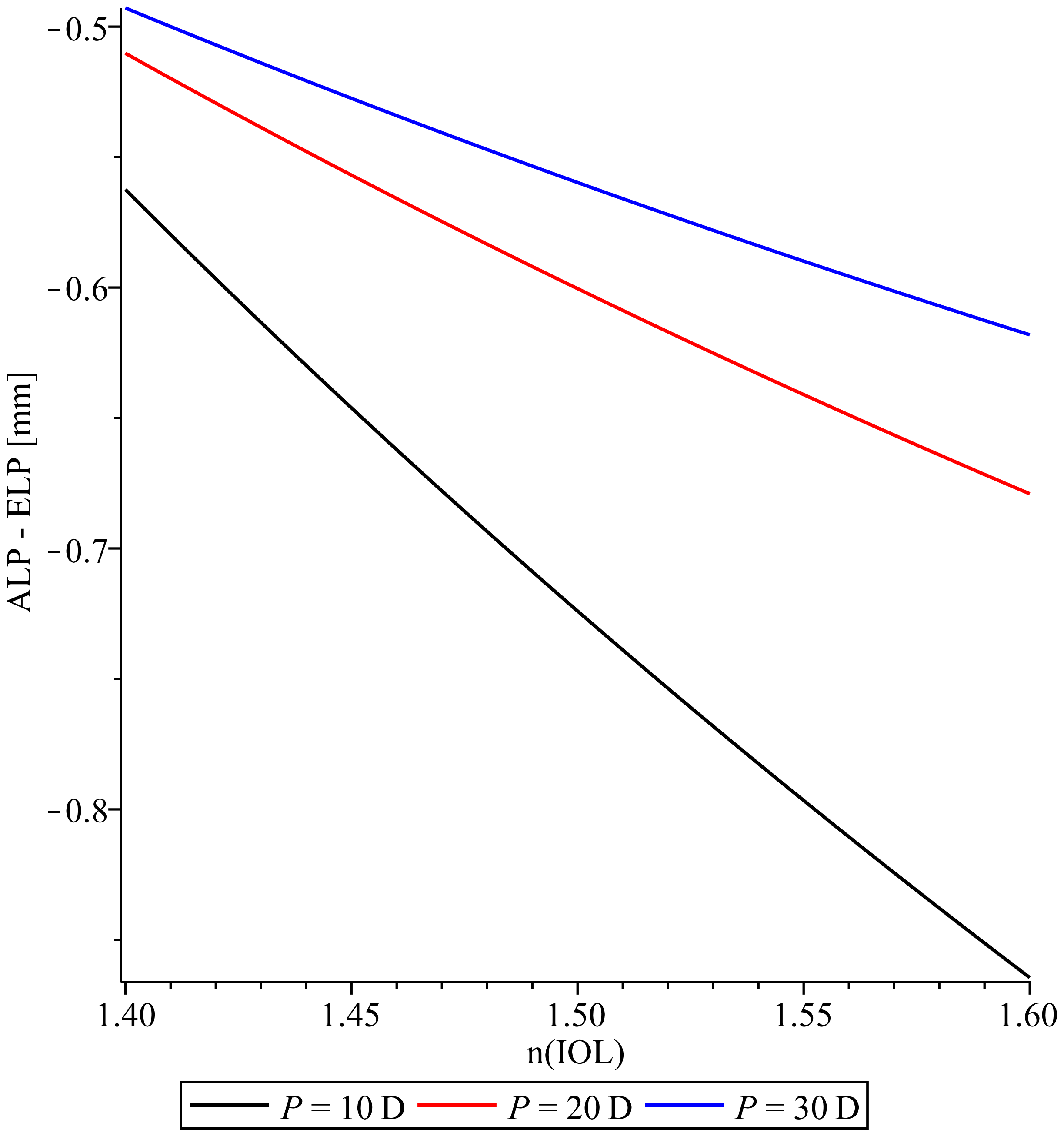


$1000 \cdot (ELPnLens(1.46) - ELPnLens(1.52))$

$$1000\, ELPnLens(1.46) - 1000\, ELPnLens(1.52) \quad \textbf{(1)}$$
